# Unusual reactions of diazocarbonyl compounds with α,β-unsaturated δ-amino esters: Rh(II)-catalyzed Wolff rearrangement and oxidative cleavage of N–H-insertion products

**DOI:** 10.3762/bjoc.12.180

**Published:** 2016-08-25

**Authors:** Valerij A Nikolaev, Jury J Medvedev, Olesia S Galkina, Ksenia V Azarova, Christoph Schneider

**Affiliations:** 1 Saint-Petersburg State University, Universitetskiy prosp. 26, St.-Petersburg 198504, Russia; 2 Universität Leipzig, Institut für Organische Chemie, Johannisallee 29, D-04103 Leipzig, Germany

**Keywords:** diazo compounds, N–H-insertion, oxidation cleavage, transition-metal-catalyzed reactions, Wolff rearrangement

## Abstract

Rh(II)-сatalyzed reactions of aroyldiazomethanes, diazoketoesters and diazodiketones with α,β-unsaturated δ-aminoesters, in contrast to reactions of diazomalonates and other diazoesters, give rise to the Wolff rearrangement and/or oxidative cleavage of the initially formed N–H-insertion products. These oxidation processes are mediated by Rh(II) catalysts possessing perfluorinated ligands. The formation of pyrrolidine structures, characteristic for catalytic reactions of diazoesters, was not observed in these processes at all.

## Introduction

Transition-metal-catalyzed reactions of diazocarbonyl compounds (DCC) with different organic substrates comprise a powerful tool of organic synthesis [1–8]. Of prime importance was found to be the ability of reactive intermediates generated from diazo compounds (ammonium, oxonium, C=X-ylides and others) to react with a variety of electrophiles/nucleophiles yielding complex and challenging organic molecules from relatively straightforward initial compounds [9–10]. The research group by Hu and co-workers elaborated recently a diversity of multicomponent reactions, which includes trapping of onium ylides by different electrophiles, such as activated С=С, С=O, C=N and other bonds [9]. A plethora of works aimed at realization of this “metal–carbene” methodology appeared in the last few years. Thus a diastereoselective approach to the synthesis of indolines via intramolecular trapping of ammonium ylides with ketones [11] and double bonds [12] was developed. C. J. Moody and co-workers elaborated an efficient way for the synthesis of pyrrolidines by trapping of ammonium ylides with ketones [13], whereas J. Sun and colleagues showed that similar reactions can be extended to compounds with triple bonds and allene fragments [14].

**Scheme 1:**
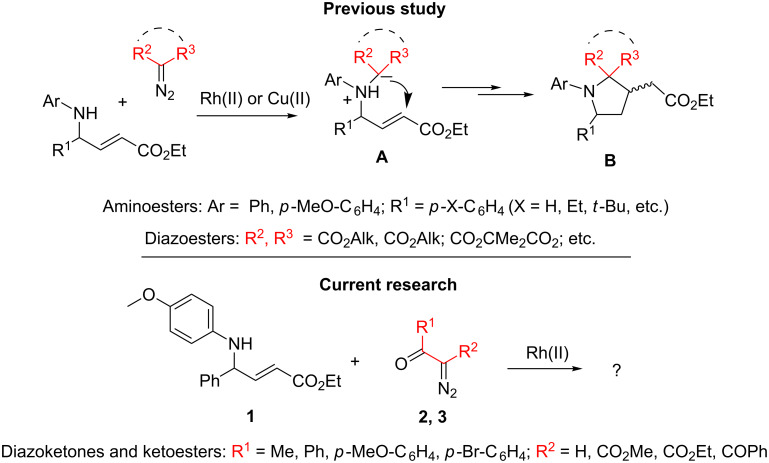
Catalytic reactions of diazocarbonyl compounds with unsaturated δ-amino esters.

Recently we have shown that the catalytic decomposition of diazomalonates and other diazoesters using Rh(II)- and Cu(II)**-**complexes in the presence of α,β-unsaturated δ-(*N*-aryl)amino esters **1** provides a good way for the synthesis of multi-functionalized *N*-arylpyrrolidines by the same “metal–carbene” methodology with yields of up to 82% [15]. The reactions occur as a domino process involving an initial formation of *N*-ylides **A** followed by the intramolecular Michael addition with the conjugated system of the amino ester to afford *N*-arylpyrrolidines **B** (Scheme 1).

It was suggested that this strategy for the synthesis of pyrrolidines could be extended to diazo compounds of other types and structures, and, as with diazoesters, multi-substituted pyrrolidines are the principal reaction products in these processes. Herein we present the main results of this study.

**Figure 1:**
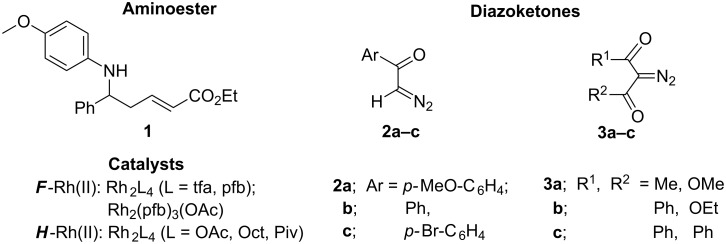
The structures of the starting compounds **1**–**3** and catalysts used in this study.

Diazocarbonyl compounds of three classes with dissimilar structures and usually different reactivity were tested in our current research: aroyldiazomethanes **2a–c**, diazoketoesters **3a,b** and diazodiketones – by the example of dibenzoyldiazomethane (**3с**, Figure 1).

The Rh(II)-сatalyzed decomposition of diazo compounds **2a–c** and **3a–c** was carried out in the presence of (*E*)-ethyl 5-((4-methoxyphenyl)amino)-5-phenylpent-2-enoate (**1**), possessing two main reaction centers – the N–H group and an activated C=C bond. To estimate the impact of the catalyst and its ligands on the efficiency of the processes studied, non-fluorinated rhodium carboxylates (Rh_2_L_4_; L = OAc, Oct, Piv) and catalysts with trifluoroacetate or perfluorobutyrate ligands [Rh_2_L_4_; L = CF_3_CO_2_ (tfa), C_3_F_7_CO_2_ (pfb)] were used in this research.

## Results and Discussion

In the beginning, catalytic reactions of aroyldiazomethanes **2a–c** were studied. Here, in all experiments formamide **4** was isolated as the major reaction product (80–99%) along with the mixtures of isomeric diarylbutenediones **5**, which are formally ‘dimers’ of intermediate aroylcarbenes (yields up to 37%) (Table 1).

**Table 1:** Rh(II)-Catalyzed reactions of diazoketones **2a–c** with aminoester **1**.

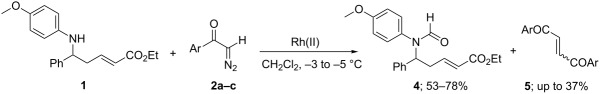		

Entry^a^	Diazoketone; Ar	Yield **4**, %^b^

1	**2a**; *p-*MeO-C_6_H_4_	78 (99)
2	**2b**; Ph	56 (92)
3	**2c**; *p*-Br-C_6_H_4_	53 (80)

^a^Reactions were carried out at −3 to −5 °C during 5 days using 3–4 equivalents of diazoketone **2**. ^b^Values shown in parentheses refer to yields related to reacted aminoester **1**.

Due to a high reactivity of diazoketones **2а–с**, catalytic reactions were carried out at −3 to −5 °C by gradually adding up to 3–4 equivalents of diazoketones **2** into reaction mixture, which was favorable for increasing the yield of the main reaction product **4** and minimization of the side process with formation of ‘dimers’ **5**. However, the complete conversion of the initial aminoester **1** was not achieved even upon using 4 equivalents of diazoketones **2** during the reaction progress. Nevertheless, the yields of formamide **4** in these reactions exceeded 80% (when calculated on the reacted aminoester **1**), whereas in the case of *p*-methoxy-substituted diazoketone **2a** the yield of the principal reaction product **4** was close to 99% (Table 1, entry 1). At the same time, no formation of the assumed pyrrolidines of type **B** was observed.

The structure of formamide **4** was reliably established using ^1^H, ^13^C and H,H-COSY NMR spectroscopy, and the composition was confirmed by HRMS.

Quite a different situation was observed with catalytic reactions of the diazoketoesters **3a,b** with aminoester **1**, which gave rise to the formation of acylamides **6a,b** in yields of 51–78% (Table 2; entries 1–3).

**Table 2:** Rh(II)-Catalyzed reactions of diazodicarbonyl compounds **3a-c** with aminoester **1**.

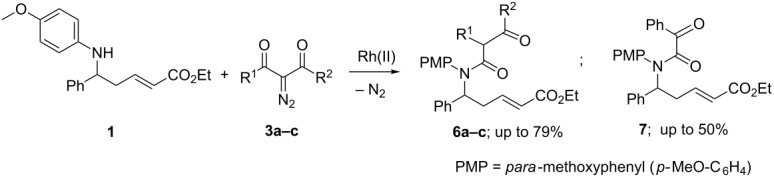					

Entry	DCC; R^1^, R^2^	Catalyst	Yield, %		

			**6a–c**	**7**	Total yield

1^a^	**3a**; Me, OMe	Rh_2_(Oct)_4_	**6a**; 51	–^b^	51
2^a^	**3b**; Ph, OEt	Rh_2_(Oct)_4_	**6b**; 70	–^b^	70
3^c^	**3b**; Ph, OEt	Rh_2_(OAc)_4_	**6b**; 78	–^b^	78
4^a^	**3c**; Ph, Ph	Rh_2_(Oct)_4_	**6c**; 79	–^b^	79
5^c^	**3c**; Ph, Ph	Rh_2_(OPiv)_4_	**6c**; 66	27	93
6^c^	**3c**; Ph, Ph	Rh_2_(OAc)_4_	**6c**; 66	15	81
7^c^	**3c**; Ph, Ph	Rh_2_(tfa)_4_	**6c**; 18	28	46
8^c^	**3c**; Ph, Ph	Rh_2_(pfb)_3_(OAc)	–	46	46
9^a^	**3c**; Ph, Ph	Rh_2_(pfb)_4_	–	50^d^	50

^a^CH_2_Cl_2_, reflux, 2–14 h; ^b^amide **7** was not identified in the reaction mixture; ^c^CH_2_Cl_2_, rt, up to 60 h; ^d^benzoic acid in a yield of 43% was isolated as a byproduct.

The highest yield with diazoketoesters **3** was obtained in reaction of benzoyldiazoacetate **3b** (70%) which was 20% more than in the case of amide **6a** obtained from the diazoacetoacetate **3a** and aminoester **1**. On usage in this reaction of Rh_2_(OAc)_4_ instead of Rh_2_(Oct)_4_ the efficiency of the process is little affected (Table 2, entries 2 and 3).

The structures of the amides **6a,b** were established by comparison of their NMR spectra with spectroscopic parameters of the same compounds obtained by a thermal decomposition of diazo compounds **3a,b** in the presence of aminoester **1** [16]. It is also quite evident that the trisubstituted acylamides **6a,b** were formed as a consequence of the initial Wolff rearrangement of diazoketoesters **3a,b** accompanied by acylation of the N–H-group of aminoester **1** with α-oxoketene formed.

When passing from diazoketoesters **3a,b** to dibenzoyldiazomethane **3с**, the formation of *N*,*N*-disubstituted 2-oxo-2-phenylacetamide **7** was observed in the yields of up to 50% (Table 2, entries 5–9) in parallel with the Wolff rearrangement product **6с** (18–79%; Table 2, entries 4–7). The structures of these compounds were established by ^1^H and ^13^C NMR, 2D NMR (H,H-COSY, HMBC, HSQC) spectra, as well as by comparison with the literature data in the case of the amide **6c** [16].

The yield of acetamide **7** was heavily dependent on the Rh(II)-catаlyst ligand nature, attaining the highest values of 46–50% when dirhodium carboxylates with perfluorinated ligands were used (Table 2, entries 7–9). Most clearly this tendency is evident when comparing the results of entries 7–9, where on passing from Rh_2_(tfa)_4_ to Rh_2_(pfb)_3_(OAc) and further to Rh_2_(pfb)_4_ the yield of phenylacetamide **7** rises from 28 up to 50%, whereas in the experiment with Rh_2_(pfb)_4_ the formation of the Wolff rearrangement product **6с** was not observed at all.

The best catalyst promoting the catalytic Wolff rearrangement in this series of experiments was found to be Rh_2_(Oct)_4_. Its application provided a means for the preparation of the amide **6с** in a yield of 79% with the total exclusion of the ‘side’ reaction product **7** (Table 2, entry 4). In all other cases the formation of a mixture of β-ketoacid amide **6с** and acetamide **7** was observed (Table 2, entries 5–9). The total yield of reaction products **6с** and **7** with Rh_2_(Piv)_4_ as the catalyst amounted up to 93% (Table 2, entry 5), evidencing on the occurrence of only two main processes at this conditions. In the experiment with Rh_2_(pfb)_4_, along with the two basic reaction products, benzoic acid was isolated as well (43%; Table 2, entry 9).

By this means during the course of Rh(II)-catalyzed reactions of aroyldiazomethanes **2a–c,** diazoketoesters **3a,b** and diazodiketone **3c** with aminoester **1**, contrary to the similar reactions of diazoesters [15], two other reaction processes were observed – the Wolff rearrangement and the assumed oxidative cleavage of the initial reaction products.

Several examples of Rh-catalyzed reactions of diazocarbonyl compounds with N–H-substrates, which are accompanied by the Wolff rearrangement with formation of corresponding amides, are known in the literature [17–25]. It is believed that these reactions follow a usual scheme of thermolysis (or photolysis) of diazocarbonyl compounds with intermediate formation of ketenes, which further acylate N-nucleophiles presented in the reaction mixture to produce the corresponding amides [20].

**Scheme 2:**
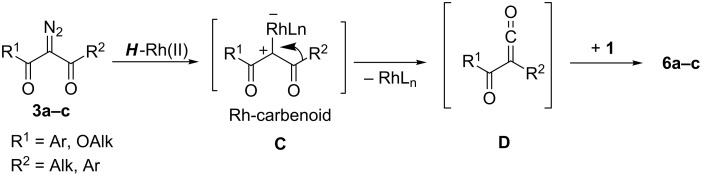
The assumed pathway for the occurance of amides **6a–c** by way of the catalytic Wolff rearrangement.

In line with these literature considerations and our results, one can suggest the next pathway for the Rh-catalyzed Wolff rearrangement (Scheme 2). Decomposition of diazocarbonyl compounds **3a–c** gives rise to generation of Rh-carbenoid **С**, ‘inside’ of which nucleophilic 1,2-migration of aryl (Ph) or alkyl (Me) group R^2^ occurs, producing α-oxoketene **D**. The latter interacts with N–H-group of the aminoester **1** to give acylamides **6a–c**.

Predominance of the Wolff rearrangement over typical carbenoid reactions (N–H-insertion, etc.) in the case of diazodicarbonyl compounds **3а–с** can be apparently explained by some sterical reasons and, first of all, by the problems associated with the approach of the N–H-group of the bulky secondary amine **1** to the electrophilic carbon atom of ***H***-Rh-carbenoid **C** (Scheme 2). Carbenoids from perfluorinated carboxylates ***F***-Rh(II) are clearly more electrophilic reagents than their ***H***-counterparts, and this enables the intermolecular process of N–H-insertion to compete successfully with the intramolecular Wolff rearrangement.

As it was shown in our study, formamide **4** and phenylacetamide **7** are formed in catalytic reactions of aroyldiazomethanes **2a–c** and dibenzoyldiazomethane **3c** with relatively high to almost quantitative yields (50–99%). It can be suggested that the appearance of amides **4** and **7** in these catalytic processes is a result of an oxidative cleavage of some reaction products, which were initially formed during the interaction of aroyldiazomethanes **2** and dibenzoyldiazomethane **3c** with aminoester **1**.

Oxidative functionalization of α-СН_2_-groups in the structure of amines is a rather familiar instrument of organic synthesis, which is widely used for the preparation of amino acids and alkaloids [26–28]. Cleavage of σ-С–С bonds in the structure of α-aminocarbonyl compounds is also a well-known transition-metal-catalyzed process, which gives rise to the occurrence of formamides and carboxylic acids [26,29]. In this case the system O_2_/TEMPO is used as a catalyst for the cleavage process, but the reaction also proceeds without the addition of TEMPO, though with lower yields [29]. Recently, communications appeared related to similar oxidation processes with participation of rhodium catalysts [30–33].

**Scheme 3:**
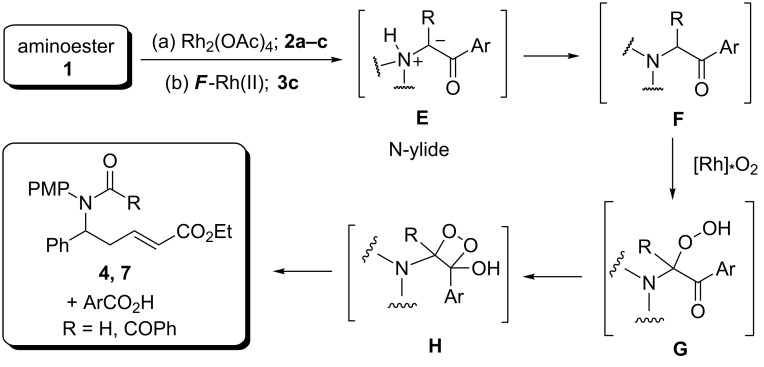
The assumed mechanism for the formation of the amides **4** and **7** during oxidative cleavage of the N–H-insertion products **F**.

Based on the literature data [29,34–35] and our current research, one can propose a mechanism for the appearance of the amides **4** and **7** during the processes studied (Scheme 3).

At first, upon catalytic decomposition of diazocarbonyl compounds **2a–c** and **3c**, N-ylide **E** is generated, stabilization of which by proton transfer produces an ordinary N–H-insertion product, α-ketoamine **F**. Similar reactions are well-known for decomposition reactions of diazocarbonyl compounds using Cu and Fe catalysts [29] and in some cases with the employment of Rh-carboxylates as well [30].

Ketoamine **F** proves to be unstable under the reaction conditions and is oxidized by a rhodium catalyst complex with oxygen producing hydroperoxide **G**, which then converts into 1,2-dioxetane **H** [30]. Subsequent cleavage of σ-С–С and О–О bonds in the structure of dioxetane **H** gives rise to the formation of amides **4** or **7** and the appropriate *рara*-substituted benzoic acid, which was isolated in several cases from reaction mixtures. A leading role in this process apparently plays the Rh-complex, since it is known that without a catalyst a cleavage of tertiary amines of this kind in the presence of oxygen does not occur [30].

It is conceivable as an alternative that the occurrence of amide **7** is derived from the oxidation of the Wolff rearrangement products **6**. However, control experiments showed that amides of β-ketoacids **6** were quite stable at the conditions of catalytic process used and their oxidative cleavage in this case did not occur.

## Conclusion

Summing up the results of catalytic reactions of aroyldiazomethanes **2**, diazoketoesters **3a,b** and diazodiketone **3c** with α,β-unsaturated δ-aminoester **1**, it should be concluded that in these reactions, unlike diazomalonates and other diazoesters, two major processes were observed – the Wolff rearrangement (with the yields of β-ketoacids amides up to 79%) and oxidative cleavage with Rh-catalysts of the initially formed N–H-insertion products to give amides **4** and **7** in yields of up to 99%. It was also demonstrated that varying the structure of the initial diazocarbonyl compounds and the nature of Rh(II)-catalyst ligands, one can change the direction of the catalytic processes studied.

## Supporting Information

File 1 Experimental details and full characterization data as well as ^1^H/^13^C NMR spectra of the new compounds.

## References

[1] Ford A, Miel H, Ring A, Slattery C N, Maguire A R, McKervey M A (2015). Chem Rev.

[2] Zhao X, Zhang Y, Wang J (2012). Chem Commun.

[3] da Silva F d C, Jordao A K, da Rocha D R, Ferreira S B, Cunha A C, Ferreira V F (2012). Curr Org Chem.

[4] Zhang Y, Wang J (2011). Eur J Org Chem.

[5] Zhang Y, Wang J (2009). Chem Commun.

[6] Zhang Z, Wang J (2008). Tetrahedron.

[7] Davies H M L, Beckwith R E J (2003). Chem Rev.

[8] Davies H M L, Denton J R (2009). Chem Soc Rev.

[9] Guo X, Hu W (2013). Acc Chem Res.

[10] Medvedev J J, Nikolaev V A (2015). Russ Chem Rev.

[11] Jing C, Xing D, Hu W (2014). Chem Commun.

[12] Jiang L, Xu R, Kang Z, Feng Y, Sun F, Hu W (2014). J Org Chem.

[13] Nicolle S M, Lewis W, Hayes C J, Moody C J (2016). Angew Chem, Int Ed.

[14] Liu K, Zhu C, Min J, Peng S, Xu G, Sun J (2015). Angew Chem, Int Ed.

[15] Medvedev J J, Galkina O S, Klinkova A A, Giera D S, Hennig L, Schneider C, Nikolaev V A (2015). Org Biomol Chem.

[16] Medvedev J J, Meleshina M V, Panikorovskii T L, Schneider C, Nikolaev V A (2015). Org Biomol Chem.

[17] Kirmse W (2002). Eur J Org Chem.

[18] Bien S, Segal Y (1977). J Org Chem.

[19] Nakano H, Ibata T (1993). Bull Chem Soc Jpn.

[20] Yañez E C, Almanza R C (2004). Rev Soc Quim Mex.

[21] Davies J R, Kane P D, Moody C J, Slawin A M Z (2005). J Org Chem.

[22] Ceccherelli P, Curini M, Marcotullio M C, Pisani E, Rosati O, Wenkert E (1997). Tetrahedron.

[23] Ronan B, Bacque E, Barriere J-C (2004). Tetrahedron.

[24] Marsden S P, Pang W-K (1999). Chem Commun.

[25] Lawlor M D, Lee T W, Danheiser R L (2000). J Org Chem.

[26] Murata S, Suzuki K, Miura M, Nomura M (1990). J Chem Soc, Perkin Trans 1.

[27] Tangari N, Tortorella V (1975). J Chem Soc, Chem Commun.

[28] Zhao Y, Ang J Q L, Ng A W T, Yeung Y-Y (2013). RSC Adv.

[29] Song R-J, Liu Y, Hu R-X, Liu Y-Y, Wu J-C, Yang X-H, Li J-H (2011). Adv Synth Catal.

[30] He J-Y, Song X-Q, Yan H, Zhong R-G (2012). J Heterocycl Chem.

[31] Doyle M P (2006). J Org Chem.

[32] Ratnikov M O, Doyle M P (2014). Mendeleev Commun.

[33] Catino A J, Forslund R E, Doyle M P (2004). J Am Chem Soc.

[34] Xing Q, Lv H, Xia C, Li F (2016). Chem Commun.

[35] Zhang C, Feng P, Jiao N (2013). J Am Chem Soc.

