# Supplementary material for: Unusual reactions of diazocarbonyl compounds with α,β-unsaturated δ-amino esters: Rh(II)-catalyzed Wolff rearrangement and oxidative cleavage of N–H-insertion products
Source: Beilstein J Org Chem. 2016 Aug 25;12:1904–10. doi: 10.3762/bjoc.12.180 (PMC5082446; doi:10.3762/bjoc.12.180)
Supplement: File 1 — Experimental details and full characterization data as well as 1H/13C NMR spectra of the new compounds. [file Beilstein_J_Org_Chem-12-1904-s001.pdf]

Supporting Information

for

Unusual reactions of diazocarbonyl compounds with α,β-unsaturated δ-amino esters: Rh(II)-catalyzed Wolff rearrangement and oxidative cleavage of N–H-insertion products

Valerij A. Nikolaev*[1], Jury J. Medvedev[1], Olesia S. Galkina[1], Ksenia V. Azarova[1] and Christoph Schneider*[2]

Address: [1]Saint-Petersburg State University, Universitetskiy prosp. 26, St.-Petersburg 198504, Russia and [2]Universität Leipzig, Institut für Organische Chemie, Johannisallee 29, D-04103 Leipzig, Germany

Email: Valerij A. Nikolaev* - valerij.nikolaev@gmail.com; Christoph Schneider* - schneider@chemie.uni-leipzig.de

*Corresponding author

Experimental details and full characterization data as well as $^{1}H/^{13}C$ NMR spectra of the new compounds

Table of contents

General information s2

Experimental part s2

Rh-Catalyzed decomposition of diazoketone **2b** in the presence of aminoester **1** s2

Rh-Catalyzed decomposition of diazoester **3a** in the presence of aminoester **1**. s3

References s7

^{1}H and ^{13}C NMR spectra of new compounds **4** and **7** s8

General information

All reactions were carried out under an argon atmosphere in solvents dried and purified before use by common methods. Monitoring of the reaction course was accomplished by thin-layer chromatography (TLC) on precoated silica gel SIL G/UV254 plates (Marchery, Nagel & Co.). Flash chromatography was performed using Merck silica gel 60, 230–400 mesh (eluent: hexane/DCM). ^{1}H and ^{13}C NMR spectra were measured using a Bruker-400 Avance NMR spectrometer. Chemical shifts are reported in ppm, and coupling constants are given in Hz.. Melting points are uncorrected. All the ESI/HR mass spectra were recorded on a «MaXis» (Bruker Daltonik GmbH). IR spectra were recorded on a Genesis ATIMattson/Unicam and Nicolet 8700. All diazo compounds **2** and **3** were prepared using previously described protocols [1-4]. Amides **6a–c** were previously obtained and characterized during the thermal decomposition of diazodiketones **3a–c** in the presence of aminoester **1** [5], but the catalytic decomposition was not previously described.

Experimental part

Rh-Catalyzed decomposition of diazoketone 2b in the presence of aminoester 1

Catalytic decomposition of diazoketone ***2b*** *in the presence of δ-aminoacid ester* ***1***: [the reaction was performed in a cryostat at −3 to −5 °C] diazoketone **2b** (53 mg, 0.37 mmol, 1 equiv) in 10 mL of CH_2Cl_2 was added dropwise during 4 h to a solution of the amino ester **1** (119 mg, 0.37 mmol, 1 equiv) in 10 mL of CH_2Cl_2 with 2 mg of $Rh_2(OAc)_4$. On the second and the fourth day one more equivalent of diazoketone **2b** and 2 mg of catalyst were added to the reaction mixture, however aminoester **1** was not fully consumed. The solvent was removed in vacuo, and the residue was separated by column chromatography (SiO_2, eluent: hexane/Et_2O 8:1 → 3:1) to obtain the starting amino ester **1** (47 mg, 39%), formamide **4** (44 mg, 56%) and a mixture of isomeric olefins **5** (97 mg).

Catalytic decomposition of diazoketone ***2a*** *in the presence of δ-amino acid ester* ***1***: [the reaction was performed in a cryostat at −3 to −5 °C] diazoketone **2a** (110 mg, 0.63 mmol, 2 equiv) in 10 mL of CH_2Cl_2 was added dropwise during 4 h to a solution of the amino ester **1** (102 mg, 0.34 mmol, 1 equiv) in 5 mL of CH_2Cl_2 with 2 mg of $Rh_2(OAc)_4$. On the second and the fourth day one more equivalent of diazoketone **2a** and 1 mg of catalyst

were added to the reaction mixture, however aminoester **1** was not fully consumed. The solvent was removed in vacuo, and the residue was separated by column chromatography (SiO_2, eluent: hexane/Et_2O 8:1 → 3:1) to obtain the starting amino ester **1** (24 mg, 23%), formamide **4** (66 mg, 78%) and a mixture of isomeric olefins **5** (78 mg).

*Catalytic decomposition of diazoketone **2c** in the presence of δ-amino acid ester **1***: [the reaction was performed in a cryostat at −3 to −5 °C] diazoketone **2c** (151 mg, 0.67 mmol, 2 equiv) in mL of CH_2Cl_2 was added dropwise during 4 h to a solution of the amino ester **1** (109 mg, 0.34 mmol, 1 equiv) in 5 mL of CH_2Cl_2 with 2 mg of $Rh_2(OAc)_4$. On the second and the fourth day one more equivalent of diazoketone **2a** and 0.5 mg of catalyst were added to the reaction mixture, however aminoester **1** was not fully consumed. The solvent was removed in vacuo, and the residue was separated by column chromatography (SiO_2, eluent: hexane/Et_2O 8 : 1 → 3: 1) to obtain the starting amino ester **1** (38 mg, 34%), formamide **4** (41 mg, 53%) and a mixture of isomeric olefins **5** (126 mg).

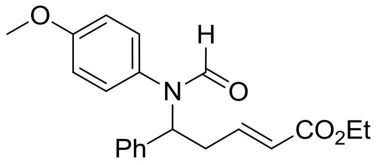

(*E*)-ethyl 5-(*N*-(4-methoxyphenyl)formamido)-5-phenylpent-2-enoate (4). Yellow oil. ^{1}H NMR (300 MHz, $CDCl_3$), *δ*, ppm.: 8.19 (s, 1H, CHO), 7.13-7.34 (m, 5H, Ph), 6.92 (dt, *J* = 15.7, 6.7 Hz, 1H, C<u>H</u>=$CHCO_2Et$), 6.75 (d, *J* = 9.0 Hz, 2H, PMP), 6.65 (d, *J* = 9.0 Hz, 2H, PMP), 5.83-6.03 (m, 2H) (CH=C<u>H</u>CO_2Et + C^5H), 4.16 (q, *J* = 7.1 Hz, 2H, OC<u>H</u>$_2$$CH_3$), 3.76 (s, 3H, OMe), 2.55-3.05 (m, 2H, CH_2), 1.26 (t, *J* = 7.1 Hz, 3H, OCH_2C<u>H</u>$_3$). ^{13}C NMR (75 MHz, $CDCl_3$), *δ*, ppm.: 166.2, 163.2 (CHO, CO_2Et), 159.5, 144.3, 138.8, 130.4, 128.6, 128.4, 128.2, 124.2, 114.3 (Ph + PMP + C^5H), 60.5, 55.5, 55.3, 33.5, 14.3 (OMe + OCH_2 + CH + CH_2 + Me); HRMS (ESI) calculated for $C_{21}H_{23}NO_4$ $[M+Na]^+$ 376.1525, found 376.1517.

<u>Rh-Catalyzed decomposition of diazoester 3a in the presence of aminoester 1.</u>

*Catalytic decomposition of diazoester **3a** in the presence of δ-amino acid ester **1***. To a mixture of δ-amino acid ester 1, 64 mg (0.197 mmol) and Rh_2Oct_4 5 mg (1 mol %) in 15 mL of absolute CH_2Cl_2 diazoester **3a** 42 mg (0.23 mmol) in 5 mL of absolute CH_2CI_2

was added. One hour after the addition of the catalyst the green reaction mixture changed to brown, after the disappearance of **3a** (after 9 h) additional 21 mg (0.115 mmol) of diazoester were added. The mixture was refluxed for 14 hours until disappearance of δ-amino ester **1** (control by TLC). After the reaction was completed, the solvent was distilled off, the residue was separated by column chromatography (25 g of silica gel, eluent: hexane/MTBE 10:1 → 0:1) to obtain amide **6a** (44 mg, 51%) as a 1.1:1 mixture of diastereomers.

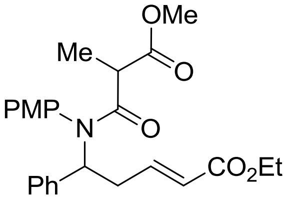

(*E*)-ethyl 5-(3-methoxy-*N*-(4-methoxyphenyl)-2-methyl-3-oxopropanamido)-5-phenylpent-2-enoate (6a). Brown oil. (*Diastereomer A*): ^{1}H NMR (300 MHz, $CDCl_3$), *δ*, ppm.:7.01-7.27 (m, 6H), 6.92-7.00 (m, 1H), 6.87-6.92 (m, 1H), 6.57-6.66 (m, 1H), 6.28 (t, *J* = 6.8 Hz, 1H), 6.12-6.14 (m, 1H), 5.93-5.97 (m, 1H) (Ph + PMP + 2CH= + C^5H), 4.17 (q, *J* = 7.1 Hz, 2H, $OC\underline{H}_2CH_3$), 3.77 (s, 3H, OMe), 3.57 (s, 3H, OMe), 3.18 (q, *J* = 7.0 Hz, 1H, C$\underline{H}$Me), 2.68-2.84 (m, 2H, CH_2), 1.31 (d, *J* = 7.1 Hz, 3H, CH$\underline{Me}$), 1.27 (t, *J* = 7.1 Hz, 3H, $OCH_2C\underline{H}_3$) (2Me). ^{13}C NMR (75 MHz, $CDCl_3$), *δ*, ppm.: 171.3, 170.7 ($2CO_2$Alk), 166.3 (O=CN), 159.7, 144.8, 138.9, 131.4, 130.3, 128.7, 128.4, 128.1, 124.0, 114.4 (2Ar + 2CH=), 60.5, 56.1, 55.5, 52.4, 44.4, 33.6, 14.4, 14.1 (2OMe+OCH_2+2CH+CH_2+2Me).

(*Diastereomer* B): IR (CCl_4, ν/cm^{-1}): 2981, 2852, 1740, 1657, 1595, 1494, 1226. ^{1}H NMR (300 MHz, $CDCl_3$), *δ*, ppm.: 7.09-7.27 (m, 6H), 6.95-7.04 (m, 1H), 6.88-6.92 (m, 1H), 6.59 (dd, *J* = 8.7, 2.9 Hz, 1H), 6.28 (t, *J* = 6.8 Hz, 1H), 6.01-6.04 (m, 1H), 5.95-6.00 (m, 1H) (Ph + PMP + 2CH= + C^5H), 4.17 (q, *J* = 7.1 Hz, 2H, $OC\underline{H}_2CH_3$), 3.78 (s, 3H, OMe), 3.68 (s, 3H, OMe), 3.16 (q, *J* = 7.0 Hz, 1H, C$\underline{H}$Me), 2.65-2.88 (m, 2H, CH_2), 1.27 (t, *J* = 7.1 Hz, 3H, $OCH_2C\underline{H}_3$), 1.23 (d, *J* = 7.1 Hz, 3H, CH$\underline{Me}$). ^{13}C NMR (75 MHz, $CDCl_3$), *δ*, ppm.: 171.3, 170.7 ($2CO_2$Alk), 166.4 (O=CN), 159.7, 144.8, 138.9, 131.6, 130.2, 128.7, 128.5, 128.1, 123.8, 114.4 (2Ar + 2CH=), 60.4, 56.0, 55.5, 52.4, 44.3, 33.6, 14.4, 14.1(2OMe+OCH_2+2CH+CH_2+2Me). HRMS (ESI) calculated for $C_{25}H_{29}NO_6$ $[M+Na]^+$ 462.1887, found 462.1888.

*Catalytic decomposition of diazoester **3b** in the presence of δ-amino acid ester **1**.*

a) To a mixture of 50 mg (0.154 mmol) of δ-amino acid ester **1**, 3 mg (1 mol %) Rh_2Oct_4 in 10 mL of absolute CH_2Cl_2, 40 mg (0.185 mmol) of diazoacetate **3b** in 5 mL of absolute CH_2Cl_2 was added, the mixture was refluxed for 11 hours until disappearance of δ-amino acid ester **1** (TLC control), 10 minutes after addition of the catalyst, the green reaction mixture changed to brown. After disappearance of **3b** (after 6 h) an additional 40 mg (0.185 mmol) of diazoester was added. After the reaction was completed, the solvent was distilled off, the residue was separated by column chromatography (25 g of silica gel, eluent: hexane/MTBE 10:1 → 0:1) to obtain amide **6b** (60 mg, 70%) as a 1:1 mixture of diastereomers.

b) An analogous reaction with $Rh_2(OAc)_4$ (1 mol %) as a catalyst was carried out at room temperature (60 h) and led to the formation of amide **6b** (67 mg, 78%).

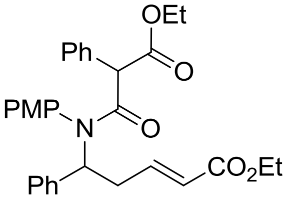

(*E*)-ethyl 5-(3-ethoxy-*N*-(4-methoxyphenyl)-3-oxo-2-phenylpropanamido)-5-phenylpent-2-enoate (6b). Brown oil. (*Diastereomer A*). ^{1}H NMR (400 MHz, $CDCl_3$), *δ*, ppm.: 7.02-7.27 (m, 12H), 6.96 (dt, *J* = 15.8, 6.6 Hz, 1H), 6.44-6.47 (m, 1H), 6.20 (t, *J* = 7.8 Hz, 1H), 5.96 (dt, *J* =15.7, 1.5 Hz, 1H), 5.64 (dd, *J* = 8.8, 2.6 Hz, 1H) (2Ph + PMP + C^{5}H + 2CH=), 4.39 (s, 1H, C<u>H</u>CO_2Et), 4.18 (q, *J* = 7.1 Hz, 2H, OC<u>H</u>$_2CH_3$), 4.17 (q, *J* = 7.2 Hz, 2H, OC<u>H</u>$_2CH_3$), 3.79 (s, 3H, OMe), 2.70-2.89 (m, 2H, CH_2), 1.28 (t, *J* = 7.1 Hz, 3H, OCH_2C<u>H</u>$_3$), 1.25 (t, *J* = 7.1 Hz, 3H, OCH_2C<u>H</u>$_3$). ^{13}C NMR (75 MHz, $CDCl_3$), δ, ppm.: 169.0, 168.4 (2CO_2Et), 166.4 (O=CN), 159.7, 144.7, 138.6, 133.5, 132.5, 131.9, 129.7, 129.6, 128.6, 128.3, 127.8, 123.9, 114.2, 114.0 (3Ar + 2CH=), 61.6, 60.4, 56.6, 56.0, 55.5, 33.6, 14.4, 14.2 (OMe + 2OCH_2 + 2CH + CH_2 + 2Me).

(*Diastereomer B*). IR (film, ν/cm^{-1}): 2980, 2872, 1751, 1719, 1654, 1605, 1583, 1509, 1296, 1273, 1250. ^{1}H NMR (400 MHz, $CDCl_3$), *δ*, ppm.: 7.11-7.27 (m, 10H), 6.87 (dt, *J* = 15.8, 6.6 Hz, 1H), 6.74 (dd, *J* = 8.7, 3.0 Hz, 1H), 6.57-6.64 (m, 2H), 6.32 (t, *J* = 7.8 Hz, 1H), 6.21 (dd, J = 6.9, 3.4 Hz, 1H), 5.86 (dt, *J* = 15.7, 1.5 Hz, 1H) (2Ph + PMP + C^{5}H +

2CH=), 4.34 (s, 1H, $C\underline{H}CO_2Et$), 4.15 (q, J = 7.2 Hz, 2H, $OC\underline{H}_2CH_3$), 4.14 (q, J = 7.1 Hz, 2H, $OC\underline{H}_2CH_3$), 3.78 (s, 3H, OMe), 2.65-2.68 (m, 2H, CH_2), 1.26 (t, J = 7.1 Hz, 3H, $OCH_2C\underline{H}_3$), 1.20 (t, J = 7.1 Hz, 3H, $OCH_2C\underline{H}_3$). ^{13}C NMR (75 MHz, $CDCl_3$), δ, ppm.: 168.9, 168.6 ($2CO_2Et$), 166.2 (O=CN), 159.7, 144.5, 138.7, 133.7, 132.0, 129.8, 129.6, 128.7, 128.4, 128.1, 127.9, 123.9, 114.3, 113.9 (3Ar + 2CH=), 61.5, 60.4, 56.8, 56.1, 55.5, 33.6, 14.4, 14.1(OMe + $2OCH_2$ + 2CH + CH_2 + 2Me). HRMS (ESI) calculated for $C_{31}H_{33}NO_6$ $[M+Na]^+$ 538.2206, found 538.2223.

Catalytic decomposition of diazodiketone ***3c*** *in the presence of δ-amino acid ester* ***1*** *(General procedure):* A solution of 60 mg of the amine **1** (0.18 mmol, 1 equiv), 93 mg of diazodiketone **3c** (0.37 mmol, 2 equiv) and rhodium catalyst [2 mol % of Rh_2L_4: L = OAc, Oct, OPiv, tfa, pfb; $Rh_2(pfb)_3(OAc)$] in 5 mL of DCM was stirred at room temperature for 48 hours [for L = tfa, OAc, OPiv and $Rh_2(pfb)_3(OAc)$] and boiled for 2 hours [for L = Oct, pfb], after which the solvent was distilled off. The composition of the reaction mixture was further analyzed by NMR spectroscopy. The major products were β-ketoamide **6c** (0–79%) and 2-oxo-2-phenylacetamide **7** (0–50%). Products were isolated by column chromatography (SiO_2, eluent: hexane/acetone 15:1 → 1:1).

Catalyst [T °C, t h]; Yield (**6c**, **7**):

- $Rh_2(Oct)_4$ [40 °C, 2 h]; 78 mg **6c** (79%).
- $Rh_2(OPiv)_4$ [25 °C, 48 h]; 65 mg **6c** (66%), 22 mg **7** (27%).
- $Rh_2(OAc)_4$ [25 °C, 48 h]; 65 mg **6c** (66%), 12 mg **7** (15%).
- $Rh_2(tfa)_4$ [25 °C, 48 h]; 18 mg **6c** (18%), 23 mg **7** (28%).
- $Rh_2(pfb)_3(OAc)$ [25 °C, 48 h]; 38 mg **7** (46%).
- $Rh_2(pfb)_4$ [40 °C, 2 h]; 41 mg **7** (50%).

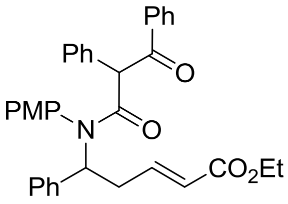

(*E*)-ethyl 5-(*N*-(4-methoxyphenyl)-3-oxo-2,3-diphenylpropanamido)-5-phenylpent-2-enoate (6c). Brown oil (1.3 : 1 mixture of diastereomers). IR (CCl_4, ν/cm^{-1}): 1717, 1684, 1648, 1510, 1322, 1252. ^1H NMR (400 MHz, $CDCl_3$), δ, ppm.: 7.58-7.69 (m, 2H), 7.40-7.51 (m, 1H), 7.02-7.34 (m, 12H), 6.87-7.00 (m, 1H), 6.57-6.79 (m, 2H), 6.49 (t, J = 2.8 Hz 1H), 6.20-6.45 (m, 1H), 5.75-6.15 (m, 2H) (3Ph + PMP +

C^5H + 2CH=), 5.27, 5.19 (s, 1H, CHCOPh), 4.09-4.23 (m, 2H, OCH_2CH_3), 3.69, 3.67 (s, 3H, OMe), 2.48-3.10 (m, 2H, CH_2), 1.28, 1.26 (t, J = 7.2 Hz, 3H, OCH_2CH_3). ^{13}C NMR (101 MHz, $CDCl_3$), δ, ppm.: 193.9, 193.7, 168.7, 168.5(COPh + CO_2Et), 166.2, 166.0 (O=CN), 159.5, 159.4, 144.5, 144.4, 138.8, 138.2, 136.2, 136.0, 133.8, 133.6, 132.8, 132.6, 132.2,131.4, 131.3, 129.8, 129.6, 129.2, 128.6, 128.5, 128.3, 128.3, 128.3, 128.2, 128.1, 128.1, 128.0, 127.9, 127.6, 127.6, 123.9, 123.8, 114.5, 114.2, 113.8, 113.6 (4Ar + 2CH=) , 60.2, 60.2, 59.9, 59.8, 56.7, 55.6, 53.4, 55.3, 33.7, 33.0, 14.2, 14.1 (OMe + OCH_2 + 2CH + CH_2 + Me. HRMS (ESI) calculated for $C_{35}H_{33}NO_5$ $[M+Na]^+$ 570.2256, found 570.2269.

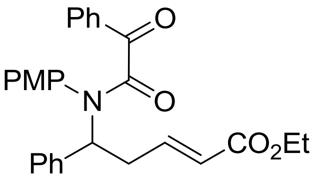

(*E*)-ethyl 5-(*N*-(4-methoxyphenyl)-2-oxo-2-phenylacetamido)-5-phenylpent-2-enoate (7). Brown oil. ^1H NMR (400 MHz, $CDCl_3$), δ, ppm.: 7.74 (dd, J = 8.4, 1.3 Hz, 2H), 7.20-7.59 (m, 10H), 7.04-7.15 (m, 1H), 6.50-6.60 (m, 2H), 6.35 (dd, J = 9.3, 6.5 Hz, 1H), 6.07 (dt, J = 15.8, 1.5 Hz, 1H) (2Ph + PMP + C^5H + 2CH=), 4.25 (q, J = 7.1 Hz, 2H, OCH_2CH_3), 3.67 (s, 3H, OMe), 2.64-3.02 (m, 2H, CH_2), 1.32 (t, J = 7.1 Hz, 3H, OCH_2CH_3). ^{13}C NMR (101 MHz, $CDCl_3$), δ, ppm.: 190.7 (PhCO), 167.7 (CO_2Et), 166.3 (O=CN), 159.8, 144.6, 138.2, 134.3, 133.7, 132.5, 129.5, 129.1, 128.9, 128.8, 128.6, 127.5, 124.6, 114.0 (3Ar + CH=), 60.7, 56.5, 55.5, 33.5, 14.5 (OMe + OCH_2 + CH + CH_2 + Me). HRMS (ESI) calculated for $C_{28}H_{27}NO_5$ $[M+Na]^+$ 480.1787, found 480.1791.

References

1. Regitz, M.; Maas, G. *Diazo compounds: properties and syntheses*; Academic Press: Orlando, **1986**; 608 p.
2. Popic, V. V.; Korneev, S. M.; Nikolaev, V. A.; Korobitsyna, I. K. *Synthesis* **1991**, 195–198. doi: 10.1055/s-1991-26416
3. Nikolaev, V. A.; Shevchenko, V. V.; Platz, M. S.; Khimich, N. N. *Russ. J. Org. Chem.* **2006**, *42*, 815–827. doi: 10.1134/S1070428006060029
4. Muthyala, M. K.; Choudhary, S.; Kumar, A. *J. Org. Chem.* **2012**, *77,* 8787–8791. doi: 10.1021/jo301529b
5. Medvedev, J. J.; Meleshina, M. V.; Panikorovskii, T. L.; Schneider, C.; Nikolaev, V. A. *Org. Biomol. Chem.* **2015**, *13,* 9107–9117. doi: 10.1039/c5ob01197c

^{1}H and ^{13}C NMR spectra of new compounds 4 and 7

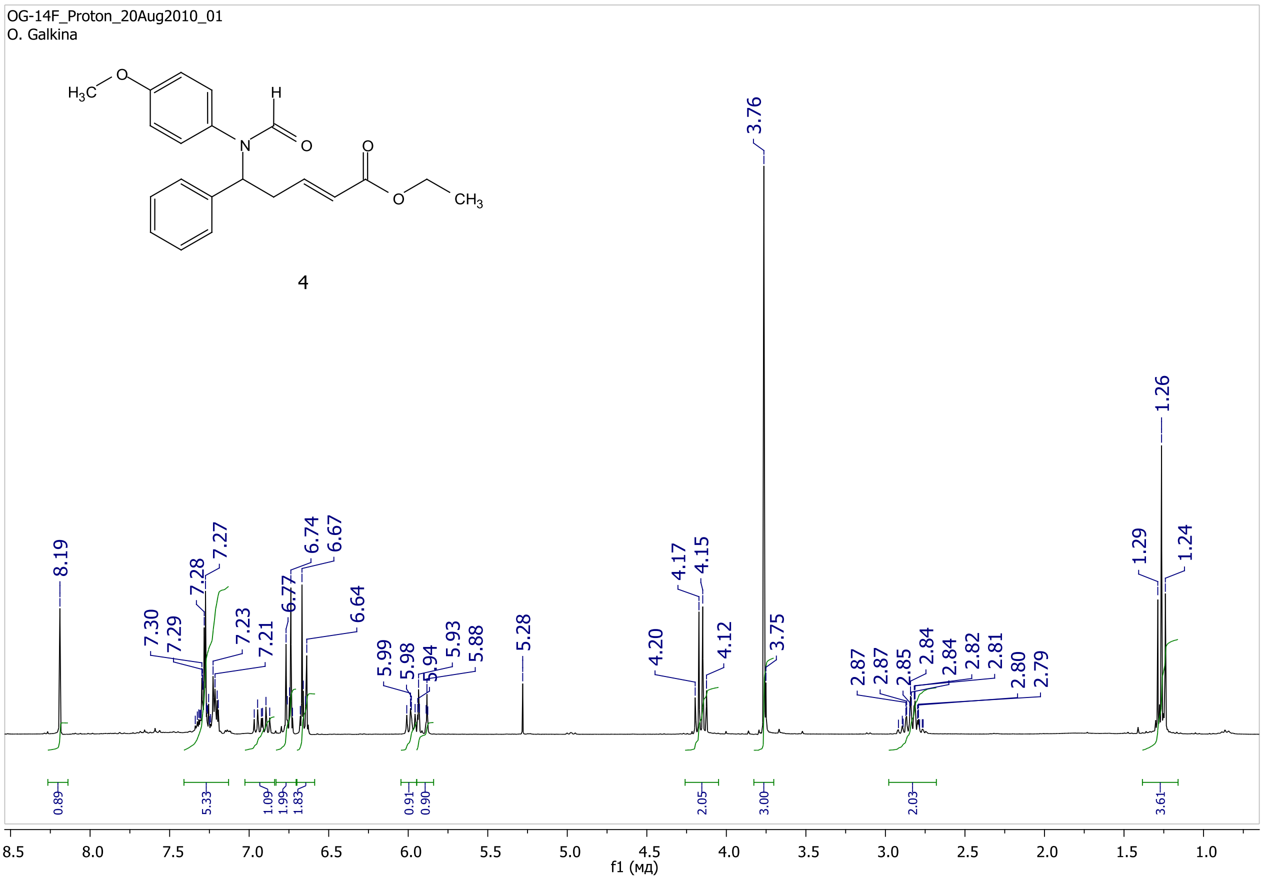

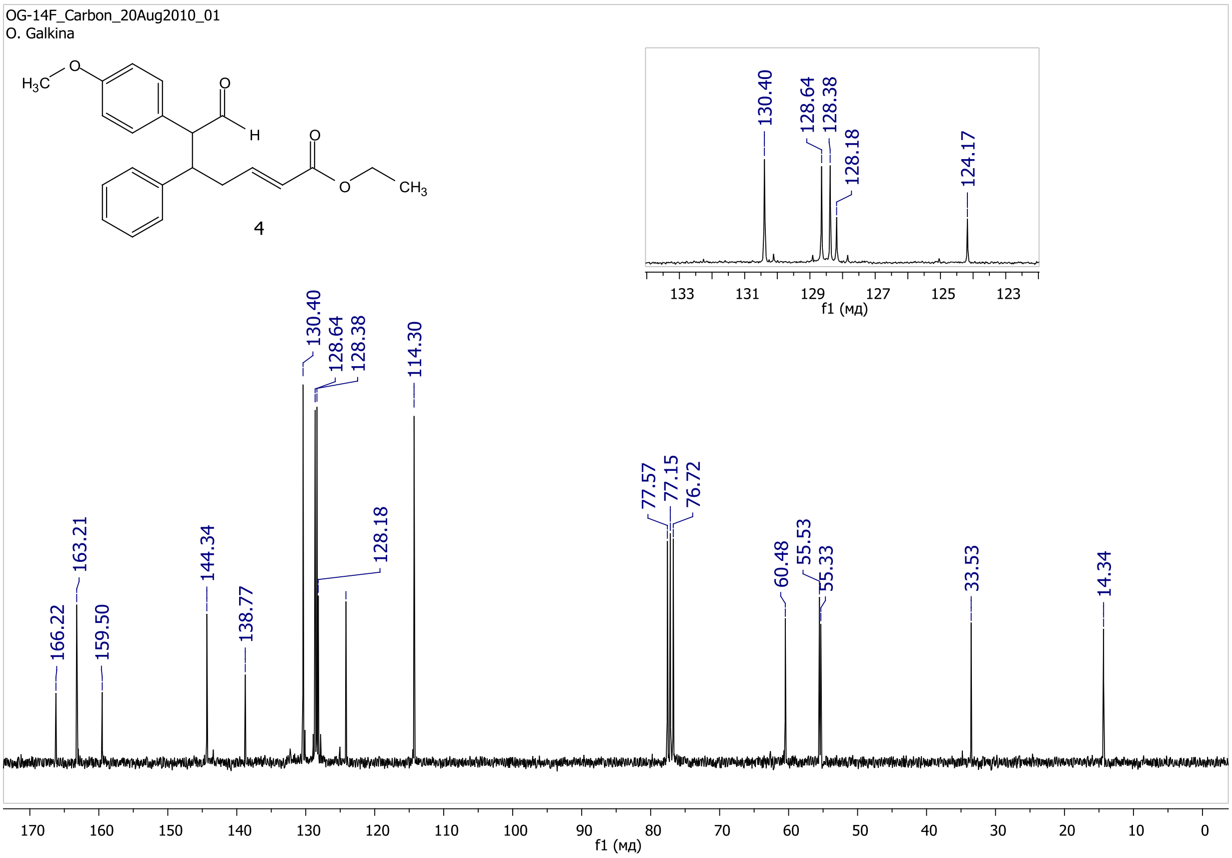

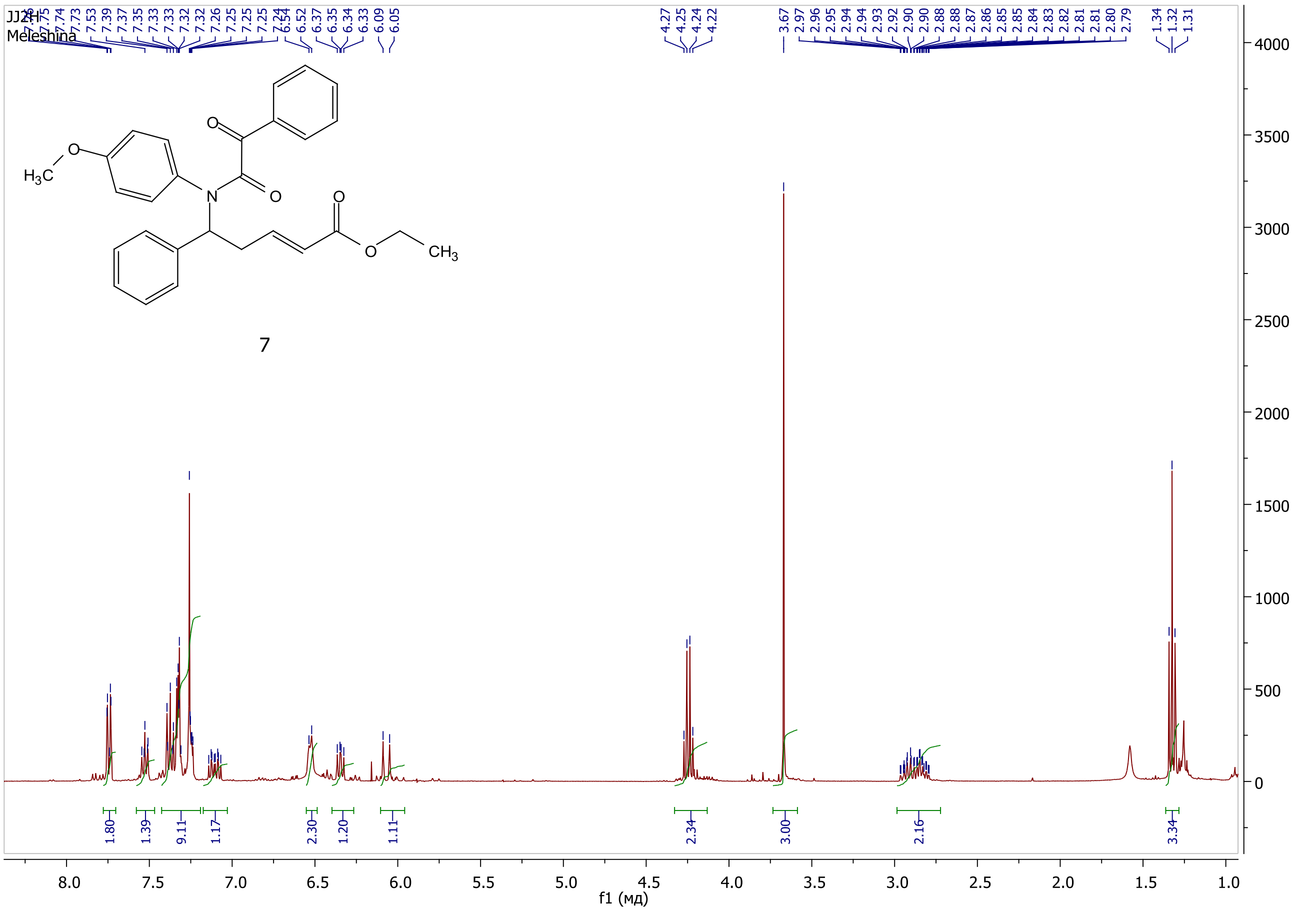
JJ2H
Meleshina
7
1.80
1.39
9.11
1.17
2.30
1.20
1.11
2.34
3.00
2.16
3.34
f1 (мд)

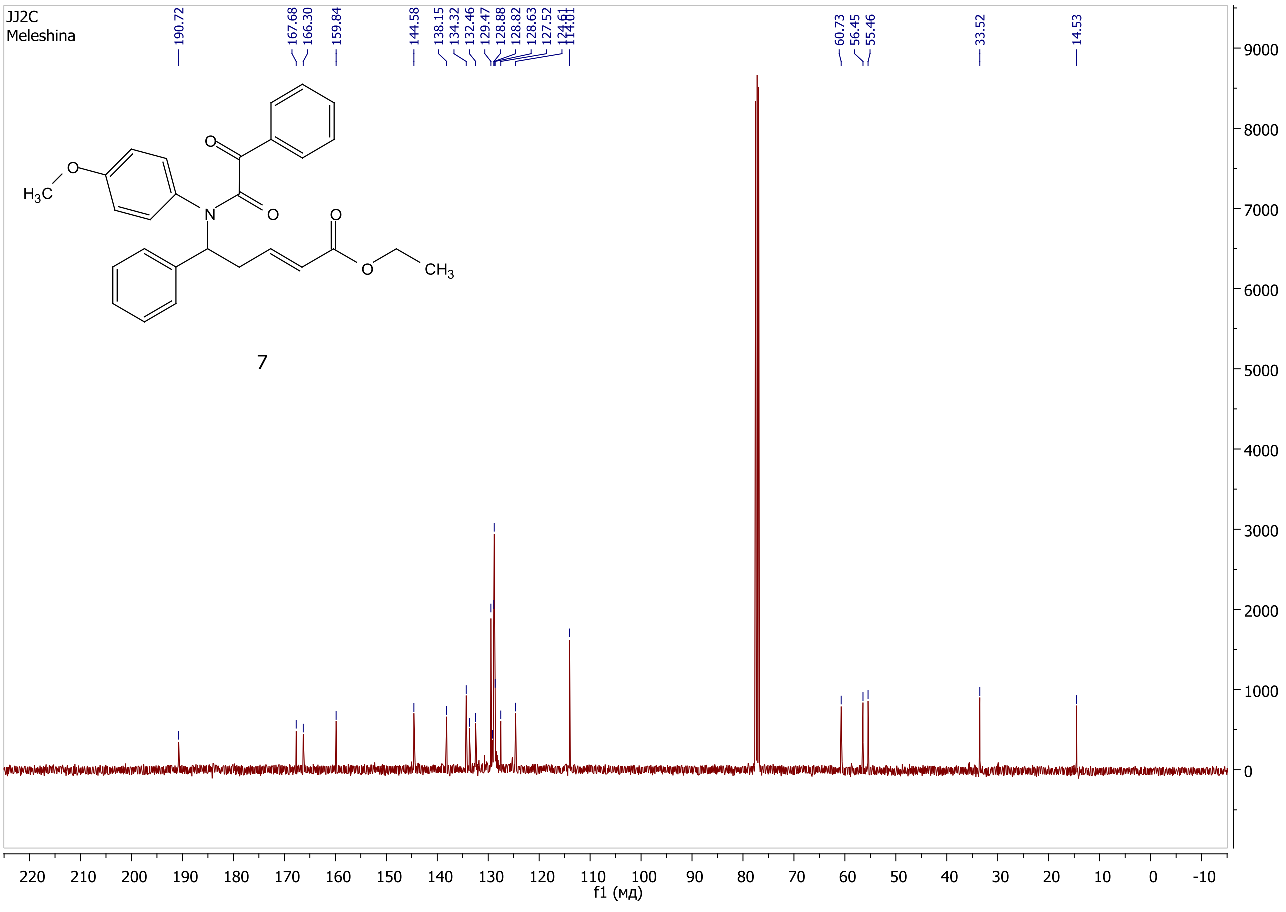
JJ2C
Meleshina
190.72
167.68
166.30
159.84
144.58
138.15
134.32
132.46
129.47
128.88
128.82
128.63
127.52
124.61
114.01
60.73
56.45
55.46
33.52
14.53
7
f1 (мд)
